# Re: The role of transperineal ultrasound in the evaluation of stress urinary incontinence

**DOI:** 10.1590/S1677-5538.IBJU.2020.1100.1

**Published:** 2022-01-10

**Authors:** Luis Augusto Seabra Rios

**Affiliations:** 1 Hospital Albert Einstein Departamento de Urologia São Paulo SP Brasil Departamento de Urologia, Hospital Albert Einstein, São Paulo SP, Brasil

*To the editor,*

Radiological evaluation of stress urinary incontinence in women (SUI) began in the last century and is performed in order to classify grade and severity of urinary leakage and better understanding of the pathology (1). Several studies validated the use of image exams regarding position and mobility of the bladder neck and proximal urethra, and correlation of hypermobility with SUI (2). However, normal values of bladder neck mobility were never defined with certainty due to great variability in young nulliparous women (3).

Many series were published discussing the use of perineal ultrasound to evaluate SUI, but in many studies, it was observed lack of uniformity of techniques and methods, preventing definition and standardization of evaluated parameters and determination of reliable evidence (4-6).

The referred study presents several virtues, including the prospective characteristic, inclusion of control group, clear definition of studied populations, use of specific and validated questionnaires, careful description of examined ultrasonographic parameters and comparison with the classic method (Q tip test), that evaluates urethral mobility. This methodology leads to clear and specific results, preventing confounders factors such and pelvic prolapse and neurological diseases (7).

The authors evaluated, other than classical determination of urethral angle, grade of descent of bladder neck and posterior urethral-vesical angle, also the grade of urethral rotation caused by the increase of abdominal pressure during Valsalva maneuver. This last parameter identifies one of the many pathological factors of SUI, the mobility and posterior-inferior rotation of proximal urethra during stress maneuvers. The use of regression analysis allowed the identification of the grade of mobility of the bladder neck as independent predictor factor of SUI. The study data also allowed the calculation of cut-off values of SUI in relation to the grade of bladder neck mobility, with high sensitivity and specificity.

Although evaluation and quantification of urethral mobility is necessary in patients with urinary incontinence, the relevance of this data must be balanced in the clinical set, since this factor is only one of the many components of pathology of SUI. Sphincter function is complex and its intrinsic deficiency is not necessarily related to urethral hypermobility or dystopia Therefore, it is important to highlight that perineal ultrasound, that however may provide accurate data regarding urethral position and mobility, does not inform precise functional data related to sphincter activity, frequently harmed in patients with SUI.

The good correlation of ultrasound data and Q tip-test must be taken into consideration when aspects such as costs and availability of ultrasound as propaedeutic method in patients with SUI are present.

The Author
